# Evidence-Based Updates to Thrombectomy: Targets, New Techniques, and Devices

**DOI:** 10.3389/fneur.2021.712527

**Published:** 2021-09-09

**Authors:** Leonard L. L. Yeo, Mingxue Jing, Pervinder Bhogal, Tianming Tu, Anil Gopinathan, Cunli Yang, Benjamin Y. Q. Tan, Fabian Arnberg, Ching-Hui Sia, Staffan Holmin, Tommy Andersson

**Affiliations:** ^1^Yong Loo Lin School of Medicine, National University of Singapore, Singapore, Singapore; ^2^Division of Neurology, Department of Medicine, National University Health System, Singapore, Singapore; ^3^Department of Neuroradiology, St. Bartholomew's and the Royal London Hospital, London, United Kingdom; ^4^Department of Neurology, National Neuroscience Institute, Singapore, Singapore; ^5^Department of Diagnostic Imaging, National University Health System, Singapore, Singapore; ^6^Department of Clinical Neuroscience, Karolinska Institutet and Department of Neuroradiology, Karolinska University Hospital, Stockholm, Sweden; ^7^Department of Cardiology, National University Heart Centre, Singapore, Singapore; ^8^Department of Medical Imaging, AZ Groeninge, Kortrijk, Belgium

**Keywords:** acute stroke, ischaemic, thrombectomy, stent retriever, aspiration, devices, reperfusion

## Abstract

Endovascular thrombectomy (EVT) has been validated in several randomized controlled trials in recent years for its efficacy in the treatment of acute ischemic strokes (AIS) and is now the standard of care according to international guidelines. However, in about 20% of EVT procedures, recanalization is not achieved, and over 50% of patients who undergo EVT still do not have good functional outcome. In this article, we provide an extensive review of the latest evidence and developments in the field of EVT, with particular focus on the factors that improve patient outcomes. These factors include new and adjunctive techniques such as combination of direct aspiration and stent retriever, intra-arterial urokinase or 2b/3a inhibitors, rescue stenting, as well as novel devices including balloon guide catheters and the newer generations of aspiration catheters and stent retrievers. We also examined the latest notion of using first-pass effect (FPE) as the target to achieve during EVT, which has been associated with an improved functional outcome. While the field of EVT has been rapidly evolving, further research is required in specific AIS patient populations such as those with large ischemic core, late presentation beyond 24 h, posterior circulation strokes, and with distal medium vessel occlusion or tandem lesions to better assess its efficacy and safety.

## Introduction

In recent years, several randomized clinical trials in acute ischemic stroke (AIS) have validated and cemented the efficacy of endovascular thrombectomy (EVT) in proximal anterior circulation occlusions (1–6). This revolutionary treatment modality has now emerged as the standard of care in international guidelines and is considered level 1 class A evidence (7, 8). Although EVT has strong evidence for the treatment of AIS, it is unable to achieve recanalization in approximately 20% of AIS patients (9, 10). Moreover, up to half of the patients who undergo EVT still do not have a good functional outcome at 3 months, and this includes patients who do have good reperfusion (6). Nonetheless, the field continues to evolve rapidly, with constant innovations. In this review, we attempt to summarize the latest evidence-based developments to the field of EVT in the context of AIS, in an attempt to determine what will allow us to improve patient outcomes.

## Targets

### eTICI as Measurement of Success in Thrombectomy

The initial measure of EVT success was extrapolated from cardiology radiological results with the thrombolysis in myocardial infarction score (TIMI). This was quickly adapted into a more cerebral circulation-based thrombolysis in cerebral infarction (TICI) score and subsequently the modified thrombolysis in cerebral infarction score (mTICI) where a mTICI 2b or greater score, equivalent to >50% reperfusion of the affected territory, is considered a successful reperfusion for EVT (11).

**Figure 1:**
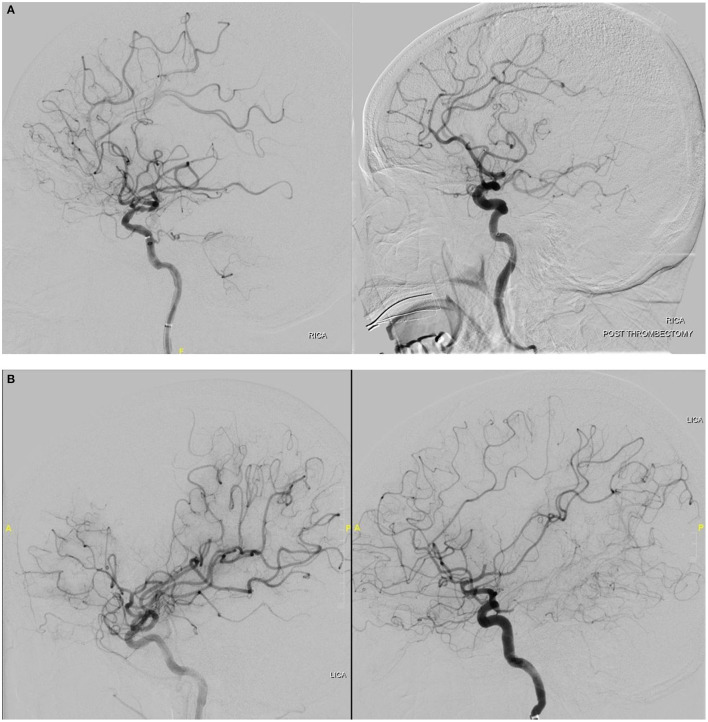
**(A)** Cerebral digital subtraction angiography showing eTICI 2b50 recanalization in a patient with right MCA occlusion. **(B)** Cerebral digital subtraction angiography showing eTICI 2b67 recanalization in a patient with left MCA occlusion.

Recently, a group of authors examined the data from the HERMES trials and proposed a revised TICI grading scale, the expanded TICI (eTICI). This is a 7-point grade from eTICI 0 to eTICI 3 which assessed the degree of reperfusion in a more quantitative manner by splitting the TICI 2b into more granular divisions (12). In brief, eTICI grade 0 is equivalent to no reperfusion or 0% filling of the downstream territory; eTICI 1 reflects thrombus reduction without any reperfusion of distal arteries; eTICI 2a is reperfusion of less than half or 1–49% of the territory; eTICI 2b50 is 50–66% reperfusion (Figure 1A), eTICI 2b67 is 67–89% reperfusion, exceeding TICI 2B but below TICI 2C (Figure 1B), eTICI 2c is equivalent to TICI 2C which represents 90–99% reperfusion or near complete; and eTICI 3 is complete or 100% reperfusion, similar to TICI 3. The authors found that after adjustment of covariates, eTICI remained an independent predictor of outcome on multivariate analysis of the mRS shift, and more importantly, adjacent categories of 2a, 2b50, and 2b67 are important distinctions with clinical implications.

In previous established consensus recommendations for reperfusion target in endovascular thrombectomy, successful reperfusion was defined as exceeding 50% of the territory (13). Now with evidence that within the >50% reperfusion category, further subdivisions into 50–66%, 67–89%, and 90–99% help to identify meaningful differences in clinical outcomes, the authors of the paper therefore proposed to adopt eTICI 2b67 as the ideal threshold for defining successful reperfusion. Moving forward, considerations should be made to adopt a more granular scale which has better prognostic value and clinical utility.

### First-Pass Recanalization: A New Target for Thrombectomy

With the advent of better equipment and improved devices for EVT, there have been increasingly better rates of successful reperfusion. Accordingly, the standard of care is constantly being re-evaluated to determine what is an achievable benchmark for AIS LVO patients. Recently, there has been a move to nominate a single pass-reperfusion standard as the target to achieve during EVT. Several studies and a recent meta-analysis have shown that there is clinical rationale behind this first-pass reperfusion with improved functional outcomes and such successful initial attempts have been termed first-pass effect (FPE) (14). In the aforementioned meta-analysis which composed 21 studies and 2,747 patients, FPE patients not only displayed better functional outcomes but also had lower mortality rates than patients who did not have FPE. Interestingly, they also showed that complete reperfusion with a single pass (FPE-mTICI 3) was associated with better 3-month outcomes compared with FPE-mTICI 2B (mRS 0–2, 66 vs. 46%; OR, 0.46; 95% CI, 0.037–0.57), better mortality rates (8 vs. 14%) and less intracranial hemorrhage (22 vs. 31%). However, it should be said that while FPE is desirable and clinical outcomes worsen with more attempts, the goal of thrombectomy is to recanalize the vessel, and recanalization after multiple passes is better than no recanalization at all (15).

Besides being used clinically, FPE mTICI ≥2B is increasingly being used to evaluate thrombectomy devices as a new benchmark. This is because in the latest trials, devices achieve >90% reperfusion rates, and there is very little differentiating them (16–18). A new threshold, FPE mTICI ≥2B or even FPE mTICI 2c-3, with its marked clinical improvement, is a potential way to evaluate these devices and tease apart their small differences, as we strive to achieve perfect stroke outcomes.

## Techniques

### Direct Aspiration vs. Stent Retriever: ASTER and COMPASS Trial

The two most common EVT techniques for reperfusion are *via* a stent retriever or aspiration with a large bore catheter. It remains a matter of debate as to which technique is superior, The Contact Aspiration vs. Stent Retriever for Successful Revascularization (ASTER) study was a randomized, open-label, blinded end-point superiority trial designed to address this problem. In this study, 381 patients were recruited, with 192 patients assigned first-line direct aspiration and 189 assigned to first-line stent retriever use. Successful reperfusion was achieved at similar rates with direct aspiration (85.4%) and stent retrievers (83.1%), *p* = 0.53. Nonetheless, this was a failed superiority trial that was underpowered to demonstrate a significant difference between the two techniques (17).

After the failure of the ASTER trial, the similar reperfusion rates between modalities led a North American group to change track and conduct a non-inferiority trial in 15 North American sites to once again compare the efficacy between large bore direct aspiration and stent retrievers. In this trial entitled COMPASS, 270 patients without a large early infarct core (ASPECTS > 6) and who presented within 6 h of onset were enrolled. Ultimately, 134 received direct aspiration as first-line treatment and 136 received stent retriever use as first-line treatment. Direct aspiration achieved 52% good functional outcomes at 3 months which was comparable with the 50% achieved by first-line stent retriever use and reached non-inferiority in the analysis (*p* = 0·0014) (18). This trial was the landmark trial to provide level 1 evidence in support of direct aspiration, and the study authors also discussed that even in the event of failure of direct aspiration, the large bore catheter is still at the clot face and a stent retriever can be quickly deployed over the thrombus. This resulted in the initial direct aspiration arm showing a significantly shorter procedural time than stent retriever use. There was also a mention of the additional benefit that aspiration catheters tend to be more affordable than stent retrievers. Nonetheless, the authors stressed that EVT technique should be tailored to the individual patient and clot characteristics for maximal efficacy.

### Combined Techniques

Thrombectomy in many centers are now performed with the combination of a stent retriever and a distal aspiration catheter as well as a balloon guide catheter. These range from the stent retriever-assisted vacuum-locked extraction (SAVE) technique, the BAlloon guiDe with large bore Distal Access catheter with dual aspiration with Stent Retriever as Standard (BADDASS) approach, the aspiration–retriever technique for stroke (ARTS), a stent retrieving into an aspiration catheter with proximal balloon technique (ASAP), or proximal balloon occlusion together with direct thrombus aspiration during stent retriever thrombectomy (PROTECT-PLUS) (19–24). These are several different variations on the same techniques for thrombectomy, all of which report a high rate of reperfusion, higher first-pass recanalization rates, lower number of attempts, and/or a lower rate of distal embolization. While it may be more expensive to use more equipment, this is offset by the improved first-pass recanalization rate and shorter procedure times which lead to better outcomes.

### Adjunctive Intra-Arterial Treatment: Urokinase, tPA, and 2b/3a Inhibitors

The Prolyse in Acute Cerebral Thromboembolism (PROACT) II trial is an early study conducted before the use of stent retrievers or aspiration catheters. It provided some grounds for intra-arterial infusion of recombinant pro-urokinase resulting in recanalization in approximately 2/3 of stroke patients with MCA occlusions (25). However, there was a five times greater risk of symptomatic intracranial hemorrhage (SICH) with pro-urokinase and production was stopped before it could obtain US FDA approval as a thrombolytic agent. It has since gradually fallen out of use in the USA, although it is still commonly used in other parts of the world.

The use of thrombolytic agents in conjunction with other thrombectomy methods has rarely been described. If urokinase has the ability to treat microthrombi, it may be able to improve forward flow and perfusion which in turn could contribute to a reduction in infarct size and rate of SICH (26). In one study where urokinase was used in 15 patients in conjunction with thrombectomy, reperfusion was improved in eight patients (53.3%) (27). In other studies, the risk of bleeding and SICH did not appear to be increased with adjunctive tPA or urokinase (28–31). A larger study including 100 patients out of a total cohort of 991 patients who received intra-arterial urokinase administered during mechanical thrombectomy was recently published. While patients who had unsuccessful thrombectomy were included, the most common reason for instillation of intra-arterial (IA) urokinase in this study was incomplete reperfusion, which was defined as mTICI <3 and seen in 53 (53%) patients. The 100 patients treated with IA urokinase during EVT did not demonstrate an increased risk of SICH or mortality. Interestingly in the 53 patients with incomplete reperfusion, there was a significantly higher rate of mRS 0–2 at 3 months (27). This study provides the groundwork for the potential use of urokinase in incomplete reperfusion and may help in improving the chance for reperfusion in distal eloquent occlusions or tortuous distal anatomy. Ultimately, it will need to be validated in larger studies such as the upcoming Multi-arm Optimization of Stroke Thrombolysis (MOST) study (32).

## Devices

### Balloon Guide Catheter

The balloon guide catheter (BGC) is a simple upgrade from the typical guide catheter with a large lumen (6–9F) and an inflatable balloon on the distal tip of the catheter. It is used to generate flow arrest of blood and even flow reversal when negative pressure is applied to remove emboli generated during the EVT procedure and prevent embolic complications. This has been shown *in vitro* where experiments with a vascular phantom occlusion model showed that use of a BGC resulted in 50% reduction of soft clot fragments compared with a conventional guide catheter (33). Similarly, in an animal porcine model, a BGC provided reproducible flow arrest with the balloon inflated, and this translated into reliable flow reversal with manual aspiration using a regular syringe. In comparison, manual aspiration with a syringe in a similar sized conventional guide catheter resulted in oscillatory flow or even an occluding collapse of the walls of the distal vessel (34).

The benefits of BGC thrombectomy has been shown in multiple different studies. In the investigator-initiated TRACK registry which audits the Trevo device, 536 anterior circulation stroke patients of whom 279 (52.1%) had BGC placement showed that mTICI 2b-3 scores were higher in the BGC group (84 vs. 75.5%; *p* = 0.01) with better 3 months outcomes (57 vs. 40%; *p* = 0.0004) and mortality rate (13 vs. 23%; *p* = 0.008). This was despite aspiration catheter or intermediate catheter use being more common in the non-BGC group (35). In the NASA and STRATIS registries (Systematic Evaluation of Patients Treated With Neurothrombectomy Devices for Acute Ischemic Stroke), a similar effect was seen for 3-month functional outcomes. Moreover, in these two registries, the FPE was more often seen with the use of a BGC (36, 37). A meta-analysis of studies with BGC use which included 2,022 patients showed that BGC use was indeed associated with a higher chance of FPE (OR, 2.1; 95% CI, 1.65–2.55) (38).

As there were certain challenges with compatibility of BCGs and large-bore distal aspiration catheters, the industry is keen on designing novel aspiration catheters that are compatible with their BCGs (e.g., novel 7Fr Catalyst fits into 8Fr Flowgate or Flowgate2 BGC). The ASTER trial documented a trend toward better mTICI 3 and better clinical outcomes in BGC-treated patients using direct aspiration (17). In the PROTECT trial with 200 patients, the group using both direct aspiration and a BGC had shorter procedure times (29 vs. 40 min; *p* = 0.002), higher rate of successful recanalization (100 vs. 78%; *p* = 0.001) and a higher rate of complete reperfusions (70 vs. 39%; *p* < 0.001) compared with sole distal aspiration during MT (22). However, larger studies are needed for confirmatory evidence to substantiate the benefits of BGC use in direct aspiration for EVT. Finally it must be mentioned that despite all purported benefits, there have been no randomized controlled trials conducted to confirm the benefits of BGC use. This is largely due to the large sample size required to show the difference and the funding required for such a trial (39).

### New Aspiration Catheters

**Figure 2:**
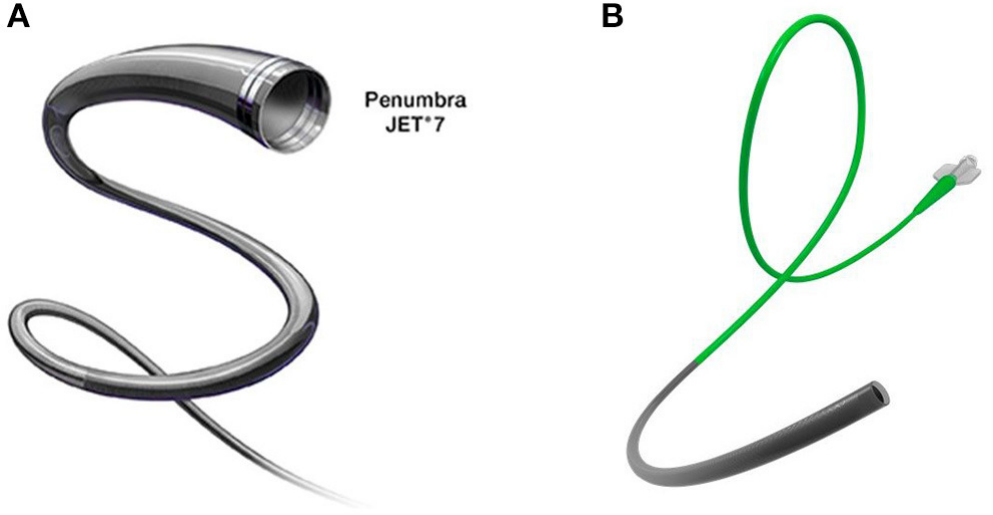
**(A)** Jet 7 aspiration catheter from Penumbra with a 0.072-in (1.83 mm) inner lumen for greater aspiration force. **(B)** React 71 aspiration catheter from Medtronic (Fridley, MN, USA) with an overlapping nitinol coil and braid design for better navigability and pushability.

The force of aspiration is directly proportional to the inner diameter (ID) of the catheter. Prior scientific examination has similarly established the powerful relationship of ID to pressure loss and flow rate in small vessels (40–42). To take advantage of this principle, three new larger bore 0.071- to 0.072-in aspiration catheters were recently introduced for stroke thrombectomy. These catheters are named the Jet 7, the Vecta 71, and the React 71 (Figures 2A,B). All three catheters are the largest bore direct aspiration catheters on the market that can fit within the current guide catheters and are able to generate a larger aspiration force. An early study looking at the navigability and efficacy of these aspiration catheters showed that they were able to reach the face of the clot in a high proportion (87%) of cases: 100% with React 71, 93% with Vecta 71, and 43% with Jet 7 (*p* = 0.002) (43). The rate of mTICI 2b-3 reperfusion was also high in all three catheters and was achieved in 92% of cases: 95% with React 71, 89% with Jet 7, and 89% with Vecta 71. These large bore catheters achieved a 39% FPE rate in this small series which provided early evidence for the use of these new catheters.

Most recently, a novel type of aspiration catheter has been introduced. The Anaconda advanced thrombectomy system (Biomed) is an aspiration catheter system that comprises a delivery catheter and a novel funnel-shaped aspiration catheter which can expand to fit the size of the vessel it is deployed in (44). The funnel shape is achieved by a stent mounted at and covered by the distal end of the catheter, which is deployed and can expand up to a maximum of 5 mm diameter, thus providing flow arrest for vessels of this size or smaller. This allows it to cause flow arrest akin to a BGC and consequently perform clot aspiration with a larger force as well as enable the catheter to accommodate larger clots and prevent distal emboli from fragments. It is, however, designed to be primarily used in conjunction with a stent retriever which is pulled inside the funnel that is subsequently closed and withdrawn. In an early study conducted by the company producing the device, the authors showed better first-pass reperfusion rates and fewer passes for revascularization compared with a BGC + stent retriever combination. Future independent studies will be needed to demonstrate the clinical efficacy of this new catheter device and its superiority to current aspiration thrombectomy technology.

The efficacy of aspiration catheters together with shorter procedural times and cost-effectiveness have pushed the development of even larger bore aspiration catheters. Currently, 8F 0.088-in (I.D.) aspiration catheters (Millipede 088, Perfuze Ltd;) and (0.088 in, Route 92 Medical, Inc., San Mateo, CA) have been proven feasible to navigate in preclinical models of the M1 segment and the basilar artery and to be superior in clot extraction compared with smaller bore catheters (45). However, in this study, there is no published clinical data on safety and efficacy profiles of these catheters from use in patients.

**Figure 3:**
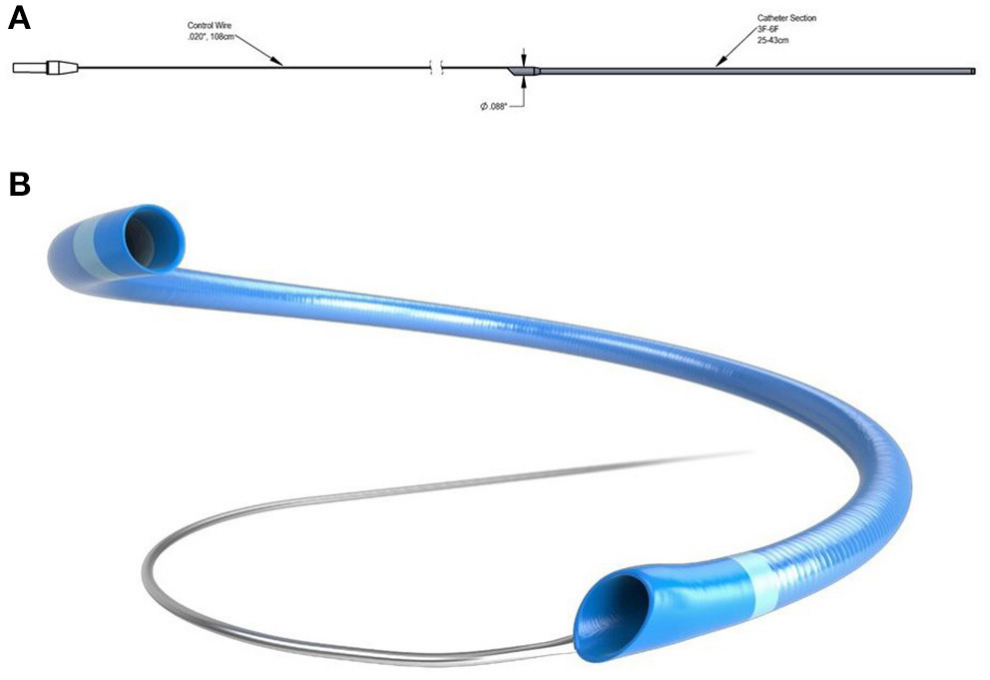
**(A,B)** 6F R4Q aspiration catheter system from MIVI (Eden Prairie, MN, USA) where the proximal portion is replaced by a wire to increase aspiration flow rates.

Aspiration catheters typically have a tapered design where the distal tip of the aspiration catheter is slimmer than the proximal shaft, to both increase suction capability while allowing for the possibility of distal access. In the end, there is a balance between the size of the inner diameter and the wall thickness of the aspiration catheter. The R^4^Q aspiration catheter (MIVI Neuroscience, Inc., Eden Prairie, MN) is designed to sidestep these limitations by substituting the proximal three-quarters of the catheter shaft with a stainless-steel wire (46). The 6F R^4^Q aspiration catheter system (Figures 3A,B) comprises a proximal pusher wire of 0.018 in and 117 cm length connected to a distal catheter of 25 cm length. It uses the guide catheter which this device is inserted into, to function as the proximal half of the catheter shaft, while the R^4^Q aspiration catheter functions as the distal half of the aspiration catheter. The use of the guide catheter shaft allows it to have a larger catheter diameter with commensurately larger aspiration power. The R^4^Q catheters come in 6F, 5F, 4F, and 3F sizes to fit to different vessel sizes. Unlike typical aspiration catheters, the suction is applied directly to the guide catheter and the R^4^Q can be retracted into the guide catheter. These properties have the potential to translate into clinical aspiration thrombectomy advances and the initial clinical experience with the R^4^Q system in a small cohort of 32 patients shows good reperfusion rates and a high rate of first-pass effect, which will need to be further validated in larger cohorts (47).

### Cyclical Aspiration Pumps

Thromboaspiration for stroke thrombectomy is typically conducted by generating a static continuous vacuum either with a pump or a large syringe. In a study on cyclical aspiration, a SOFIA Plus catheter (MicroVention Inc., Aliso Viejo, CA) had static (29 inHg) or cyclical (18–29 inHg, 0.5 Hz) aspiration employed using the digital CLEAR Aspiration System (Insera Therapeutics, Sacramento, CA) and eight thrombus aspiration experiments were conducted for each aspiration type in a flow model. The study showed that by varying the pressure dynamics through cyclical aspiration, it increased aspiration force on the occlusion as well as resulted in more successful clot clearance when compared with static aspiration (48). The cause of this improved ingestion may be due to the initial clot softening from dynamic compression or that dynamic friction is less than the static friction that occurs when the thrombus is stuck at the tip of the catheter. This was validated in a different study using various types of catheters with different inner diameters (0.054–0.088 in), where the use of cyclic aspiration (18–29 inHg, 0.5 Hz) resulted in better clot ingestion into the aspiration catheter and effectively reduced the rate of distal emboli (49). More recently, in one of the first clinical experience with cyclical aspiration for large-vessel strokes, investigators performed thrombectomy using the CLEAR™ Aspiration System (Insera Therapeutics Inc., Dallas, TX) which yielded promising results. The authors reported high rate of TICI 3 FPE, which was achieved in 68.4% of the study cohort (26 of 38 patients), near-complete reperfusion (TICI 2c/3 FPE) in 76.3% (29/38), and substantial reperfusion (TICI 2b-67/3 FPE) in 78.9% (30/38). In addition, final revascularization results for the entire cohort (in one or multiple attempts) were TICI 3 in 86.8% (33/38), TICI 2c/3 in 94.7% (36/38), TICI 2b-67/3 in 97.4% (37/38), and TICI 2b/3 in 100% (38/38) (50). The high FPE rate also translated into neurological improvement and better functional outcome with 92.1% of the cohort achieving NIHSS improvement of at least 4 points at 24 h and 81.6% having good outcomes (mRS 0–2) at discharge. There were no symptomatic intracerebral hemorrhages and at 90 days, the all-cause mortality was low at 5.3%.

### Third-Generation Stent Retrievers

The second-generation Solitaire and Trevo stent retrievers have been widely used and contributed greatly to the fact that EVT is now the standard of care for LVO AIS. However, this technology for stent retrievers has also evolved over time with a recent explosive improvement in the technology. It should be mentioned that despite this, randomized controlled trials showing an improvement in recanalization, functional outcomes, and reduced complications compared with existing stent retrievers have yet to be performed.

**Figure 4:**
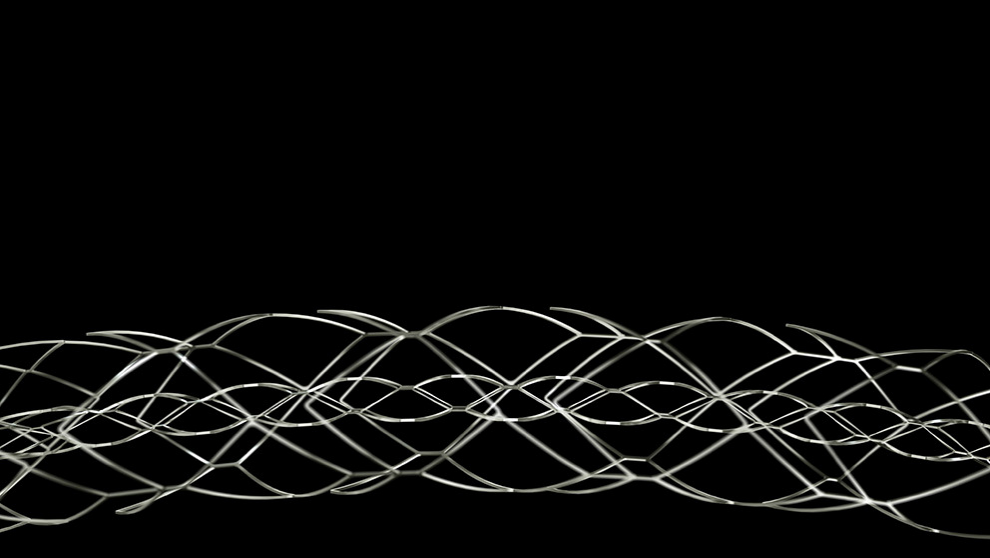
EmboTrap reperfusion device (third-generation stent retriever) from Cerenovus (Miami, FL, USA).

The EmboTrap reperfusion device (Neuravi/Cerenovus) is a third-generation stent retriever with a dual-layer structure furnished with articulating petals and a distal capture zone, supposed to enable better grip on the clot and entrapment of clot fragments generated by the EVT procedure (Figure 4).

**Figure 5:**
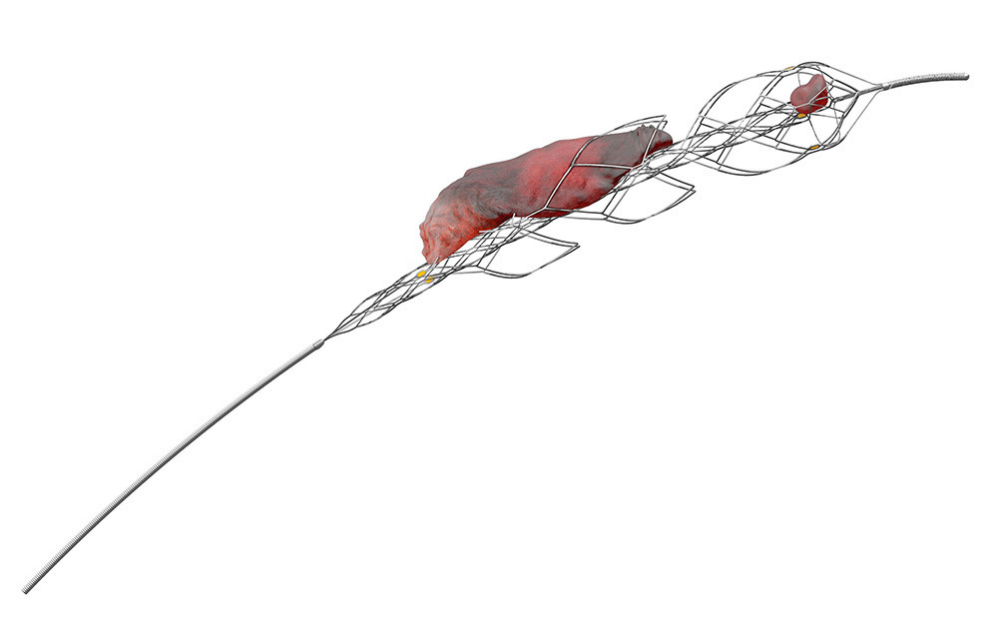
EmboTrap stent retriever shown with a clot *in situ*.

The efficacy of the Embotrap stent was validated by an open-label, single-arm, multicenter, prospective clinical trial conducted by the company which produced the stent, entitled Analysis of Revascularization in Ischemic Stroke with EmboTrap (ARISE II) which enrolled 227 patients (16). The mTICI ≥2b reperfusion within three passes was achieved in 80.2%, while the final mTICI ≥2b reperfusion rate was 92.5%. Good functional outcome of mRS 0–2 at 90 days was achieved by 67% of the cohort, with a mortality rate of 9%. Following this study, Embotrap was granted FDA approval for use in stroke thrombectomy. There are currently newer versions of the device, namely, the Embotrap 2 and Embotrap 3 in the market (Figure 5).

**Figure 6:**
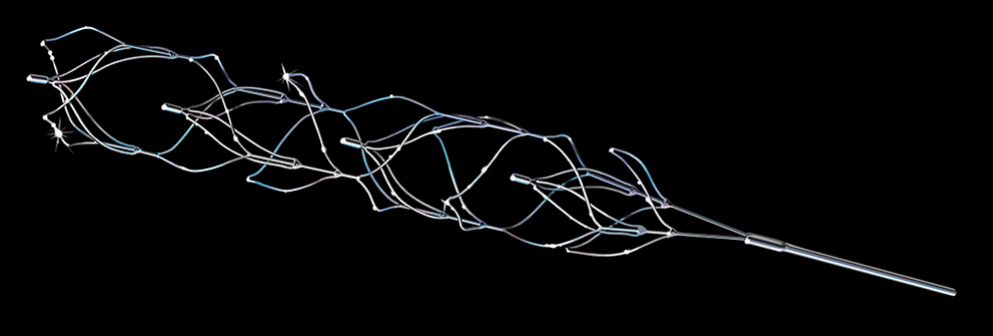
Three-dimensional revascularization device from Penumbra.

Another third-generation stent retriever is the three-dimensional (3D) revascularization device (Penumbra Alameda, CA, USA). A multicentric randomized control trial with 198 patients was performed to evaluate the safety and efficacy of this device in combination with an intermediate catheter (51). Out of the 198 recruited patients, 98 underwent thrombectomy with the 3D stent retriever (Figure 6) in conjunction with an intermediate catheter and was able to achieve mTICI 2b-3 reperfusion in 81.9% of the patients. This rate was significantly higher than the comparison arm, where direct aspiration alone with an intermediate catheter achieved only 69.8% mTICI 2b-3 reperfusion in 100 patients.

A new-generation stent retriever is the Versi, a nitinol stent retriever with two to four articulating segments expanding and reconfiguring under traction, during withdrawal, thus presumably facilitating clot trapping. An investigator-led clinical trial compared the third-generation Embotrap and Versi stents with the earlier generation Solitaire and Trevo stent retrievers (52). They employed different flow models with various degrees of tortuosity to evaluate the different stent retrievers. The authors reported that the Versi had significantly better recanalization rates than the second-generation stent retrievers, while the Embotrap also had higher recanalization rates although not reaching statistical significance. The authors were able to discern that more severe tortuosity limited the effectiveness of earlier generation stent retrievers but not effectiveness of third-generation stent retrievers. The finding could explain increased efficacy of third-generation stent retrievers. Nonetheless, this was a study performed in several flow models and remains to be validated in animal models as well as in clinical trials.

Another multisegmental stent retriever is known as the NeVa thrombectomy device. The NeVa device was designed to have an elevated radial force and has Drop Zones that help in the extraction of thrombi that adhere firmly to the artery wall. It is compatible with 0.021 in. microcatheters and is available in multiple sizes: M1-S (4 × 22 mm), M1 (4 × 30 mm), and T (4.5 × 37 mm) with the M1-S having no proximal “flow restoration zone” (53). The final segment is fashioned into a closed-ended basket, which retains the thrombus that has fallen into the Drop Zone openings. In an early single-center clinical trial with 118 patients, the rate of successful recanalization was 95.8% with first-pass mTICI 2b/3 rates achieved in 56.8% of the patients and mTICI 2c/3 rates in 44.9% of the patients. Favorable functional outcome (modified Rankin Scale 0–2) was seen in 42.4% of the population. The authors reported a 3.3% SICH rate with the rate of embolization into new territory at 1.7% (53).

Another novel clot retriever is the Tigertriever (Rapid Medical, Yokneam, Israel) equipped with a handle-controlled mechanism allowing the operator to incrementally adjust the diameter of a nitinol-braided stent as well as collapse it. The feature of diameter adjustment is hypothesized to result in better wall apposition, robust clot integration, and variable exertion of radial force in different vascular segments (54, 55). Three versions of the Tigertriever device are available: The standard version (Tigertriever) has a net length of 32 mm (unexpanded form) and can expand up to 6 mm diameter, can be delivered through a microcatheter with an internal diameter of 0.021 in. In addition, a smaller version (Tigertriever 17) has a net length of 23 mm (unexpanded form) and can be delivered through a microcatheter with an internal diameter of 0.017 in. It can expand up to 3 mm diameter. Finally, there is a new Tigertriever 13 which can fit through a 0.013-in. microcatheter. The Tigertriever device is CE approved since 2016 and completed several phase 1 trials (54–57). The investigators of the multicenter TIGER trial recently published their preliminary result. The Treatment With Intent to Generate Endovascular Reperfusion (TIGER) trial is a single-arm, prospective, multicenter trial comparing the Tigertriever to outcome in six recent pivotal studies (TREVO 2, SWIFT, MR CLEAN, ESCAPE, REVASCAT, and SWIFT PRIME) evaluating the Solitaire and Trevo stent-retriever devices. In the 160 enrolled patients treated with the Tigertriever, the primary efficacy end-point of mTICI 2b-3 reperfusion within three passes without use of rescue therapy was achieved in 84.6% in the main-study phase group compared with the 73.4% historical rate (non-inferiority *p* < 0.0001; superiority *p* < 0.01). Successful reperfusion (mTICI ≥2b) was achieved in 95.7% of the cohort and excellent reperfusion (mTICI 2c-3) in 71.8%. The primary safety composite end point rate of mortality and symptomatic intracranial hemorrhage was 18.1% compared with the 20.4% historical rate (non-inferiority *p* = 0.004; superiority *p* = 0.57). In the secondary outcome analysis, functional independence (mRS score 0–2) was attained by 58.0%, a rate superior to the 43.5% in the pooled comparator trials (*p* = 0.006) (58). The Tigertriever also appears promising in the treatment of distal, medium vessel occlusions (DMVOs) or as rescue treatment. In a cohort of 115 patients with both primary and secondary (after failed or incomplete EVT) DMVOs, as well as distal vessel thromboembolic complications during cerebral aneurysm or AVM embolization, mechanical thrombectomy performed with Tigertriever achieved successful recanalization in 86 patients (74.7%) (59).

## Adjunctive Techniques

### Rescue Stenting

**Figure 7:**
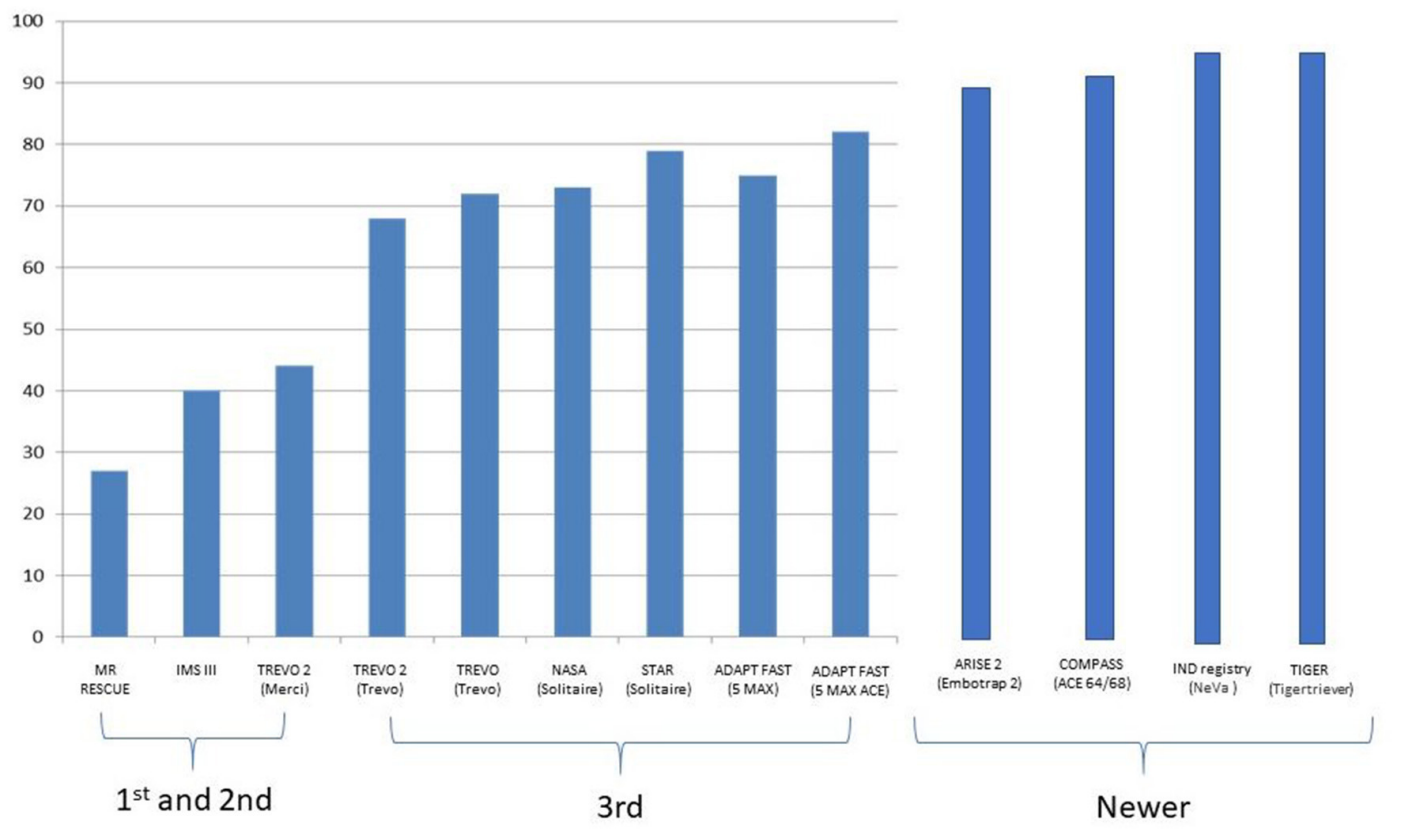
Increasing MTICI 2b-3 reperfusions rates being reported with each successive generation of devices.

While the advent of the latest stent retrievers and large bore aspiration catheters have an increase of 90% reperfusion rates in LVO thrombectomy (Figure 7), there still exists a subset of patients whereby the EVT procedure fails to recanalize the vessel. Although there are several possible factors, one of the key reasons for failure of EVT is underlying intracranial atherosclerotic stenosis (ICAS). ICAS is commonly diagnosed during the EVT procedure by repeated recanalization and then acute re-occlusion of the vessel. In a Korean series of failed EVT, this occurred in up to 77% of the patient cohort (60). There are currently no guidelines on the optimum treatment in patients who failed thrombectomy and one possible technique is to perform permanent stenting to try to salvage the situation, in an attempt to keep the vessel patent. This is termed “rescue stenting” and was shown to be effective in an initial study of 45 failed thrombectomy patients, where the 17 patients with rescue stenting had better outcomes and less thirorcococn cerebral herniation than the 28 non-stenting patient (61).

Rescue stenting was validated in a large retrospective analysis of patients from 16 Korean stroke centers (62). In this study, patients with anterior circulation LVO who failed to recanalize following EVT were split into rescue stenting and nonrescue stenting groups. Out of 148 failed EVT patients, 48 received rescue stenting while 100 did not; 31/48 rescue stenting patients (64.6%) had successful mTICI 2b-3 reperfusion with rescue stenting, while none of the 100 patients without rescue stenting achieved reperfusion. Good functional outcome at 3 months was observed in 39.6% of the rescue stenting group and in 22.0% of the non-rescue stenting group (*p* = 0.031) without an increase in SICH or mortality. Of note, in the rescue stenting group who had successful reperfusion, 54.8% achieved good outcome despite the initial EVT failure, equivalent to the functional outcomes with mTICI 2b-3 reperfusion in the initial EVT attempts of 55.4%. A meta-analysis on rescue stenting which included articles from 2015 to 2019, similarly found that in a sample of 352 patients, there was improved outcomes in the stenting arm compared with the refractory occlusion arm (OR, 2.87; 95% CI, 1.77–4.66; *p* < 0.001; *I*^2^, 0%) with reduced mortality although there was some heterogeneity between studies for mortality (OR, 0.39; 95% CI, 0.16–0.93; *p* = 0.03; *I*^2^, 43%). The rates of SICH were not significantly different between both arms (63).

In a recent multinational study on emergency rescue stenting in AIS involving seven neurovascular centers (64), good outcome was observed in 73 of 163 (44.8%) patients with recorded outcomes at 90 days. This is considerably better than the rates of 7% to 22% in cohorts with re-occlusion or persistent occlusion reports without rescue stenting. However, the rate of SICH in this analysis (11%) was higher than in the aggregated thrombectomy studies without intracranial stenting of 4.4%, and this was more common in anterior circulation occlusions than posterior circulation occlusions. A lower number of thrombectomy attempts before rescue stenting was also associated with better functional outcomes.

The choice to perform rescue stenting is difficult, as the permanent placement of a stent requires either acute glycoprotein 2b/3a inhibitors or dual antiplatelet treatment to prevent acute re-occlusion from in-stent thrombosis. In acute stroke patients with already sizable amounts of ischemic or infarcted tissue, these medications contribute to a potentially higher risk of symptomatic intracranial hemorrhage. Currently, there is little data and lack of consensus regarding antiplatelet management for intracranial stenting during thrombetomy. There is considerable variation in opinions even among the experts in this field. In an attempt to establish consensus on perioperative and postoperative antiplatelets management using the DELPHI method, a panel of 19 experts were surveyed. While the panel agreed that antiplatelet management in this setting should be standardized regardless of the size of the ischemic “core” on initial brain imaging or final perfusion result or treatment with intravenous alteplase, and that intravenous followed by oral aspirin is a possible choice, it failed to achieve consensus on several other important questions such as timing of initiation of therapy and the need for second antiplatelet agent and the choice of the second antiplatelet agent (65). Therefore, more data are needed to investigate the timing of initiation and choice of antiplatelet management in patients who undergo stenting in the setting of endovascular thrombectomy. Interestingly though, observational studies which have evaluated rescue stenting have demonstrated that the use of glycoprotein 2b/3a inhibitors did not significantly increase rates of SICH maintaining stent patency (61, 62, 66–70).

### Fibrin Capsule of a Clot Broken by the Stent Retriever Followed by 2b/3a Inhibitors

For distal clots or clots past a tortuous intracranial segment, one technique is to deploy a stent retriever over the clot followed by administration of thrombolytics or glycoprotein 2b/3a inhibitor to dissolve the clot. The stent retriever is then resheathed in the microcatheter and removed, as there is a risk of avulsion of pial perforators with withdrawal of a deployed stent retriever. A small publication of 18 cases that initially failed thrombectomy, had a Solitaire stent retriever used as such with concomitant IA tirofiban, a glycoprotein 2b/3a inhibitor, administered. It showed successful reperfusion in 14 out of 18 patients (77.7%) with good functional outcomes seen in 50% of the cases (69).

A more recent publication used a scanning electron microscope to review the structure of 199 thrombi extracted during thrombectomy procedures (71). Despite the heterogeneity of clot composition and organization, thrombi demonstrated a similar outer shell made of compacted fibrin, Von Willebrand factor, and platelets. This prevented the tPA from reaching the inner core of the thrombus, and hence preventing lysis. This study provides a potential explanation on how deploying a stent retriever is able to break the outer shell of the thrombus and expose the inner core to the 2b/3a inhibitor.

## Imminent Questions to Answer

### Large Ischemic Core

While the current guidelines are clear that EVT should be performed for patients without a large early infarct or with an ASPECTS of six or more, there is a substantial proportion of patients who present to the hospital with an AIS with a sizable ischemic core. While the evidence is not clear, these patients may still derive some benefit from EVT. Pending the outcomes of randomized controlled trials, core-lab adjudicated pooled analyses of existing studies may shed preliminary insight into this crucial question. In one such study pooling data from seven randomized trials with a total of 1,764 patients, of which 871 were in the EVT arm and 893 in the best medical treatment arm, it was demonstrated that EVT was associated with better functional outcomes across a wide range of pretreatment imaging types on ordinal shift analyses (72). This included all ASPECTS groups except for ASPECTS 0–2 where the low sample size could not demonstrate statistical significance. However, this should be interpreted with caution, as in patients with a large ischemic burden or ASPECTS 4 or less, EVT was associated with significantly more SICH. Similarly, in the secondary analysis of the Optimizing Patient's Selection for Endovascular Treatment in Acute Ischemic Stroke (SELECT) trial, EVT was associated with better functional independence (mRS 0–2) compared with medical management alone (OR, 3.27; 95% CI, 1.11–9.62; *p* = 0.03). EVT was also associated with less infarct growth, and smaller final infarct volume than medical treatment (73). Such analyses provide a strong foundation to support further investigation of the use of EVT for patients with large infarcts and poor ASPECTS at baseline. These initial results led to the ongoing SELECT-2 trial which is designed to evaluate thrombectomy compared with medical management in distal ICA and MCA M1 occlusions with a large core on either CT (ASPECTS 3–5) or advanced perfusion imaging (rCBF <30% or ADC <620 or 50 ml or more) and is expected to be completed at the end of 2021 (74). Other ongoing clinical trials, such as TENSION (NCT03094715), TESLA (NCT03805308), and IN EXTREMIS (75, 76) are also recruiting patients and will provide conclusive evidence on the use of EVT in patients with a large ischemic core at presentation.

### Clinically Mild Strokes With LVO

A different group of patients are those who present with LVOs but mild strokes clinically. These are typically defined at NIHSS threshold of <5, where the risk of the EVT procedure needs to be weighed carefully against the potential benefit. A pooled data analysis of six comprehensive stroke centers with 300 patients having LVO and NIHSS 0–5, with 80 patients undergoing EVT and 220 patients undergoing best medical therapy was published (77). Of note is that the best medical treatment group allowed for rescue EVT if there was subsequent neurological deterioration. While the groups were not similar, EVT was associated with better functional outcomes (OR, 3.1; 95% CI, 1.4–6.9) and in a propensity matched analysis, the superiority of EVT over best medical treatment persisted (84.4% vs. 70.1%; *P* = 0.03).

Recently, another meta-analysis pooled patient data from 16 centers from 2013 to 2017. This study evaluated 251 patients with LVO and mild stroke of which 138 were treated with EVT and 113 with best medical treatment. The study revealed that the 3-month functional outcomes were better in the best medical treatment group compared with the EVT group (77.4% vs. 88.5%; *p* = 0.02) (78). The rate of asymptomatic ICH was also lower in the best medical management group as compared with the EVT group (4.6% vs. 17.5%; *p* = 0.002). The two groups did not differ in the rate of reperfusion or in safety outcomes.

We look forward to upcoming RCTs to further elucidate if mild strokes with LVO should be treated *via* EVT. One of these RCTs is ENDOLOW, which investigates anterior circulation occlusions with NIHSS scores 0–5 and is enrolling patients in Canada, the USA, Germany, and Sweden (79). The IN-EXTREMIS trial also includes a substudy, which evaluates ischemic stroke patients with NIHSS <6 and LVO occlusions and similarly will be aiming to answer this important question (76).

### Very Late Presenting Patients: Acute Stroke Beyond 24 h

Evidence has recently emerged from the AURORA study, which pooled data from six randomized trials to examine effect of EVT in anterior circulation proximal LVO stroke from 6 to 24 h from time last seen well, that there is benefit of EVT in achieving reduced disability on functional outcome in terms of mRS in this group of patients, with an adjusted common odds ratio of 2.54 (95% CI, 1.82–3.54; *p* < 0.0001). In addition, the Number Needed to Treat to reduce mRS by 1 point was three patients. Furthermore, no significant differences in mortality or SICH were seen between EVT and control groups (80). This further substantiates the results of previous trials which elucidated that carefully selected patients benefit from thrombectomy up to 24 h (81, 82). However, there remains a pool of patients who present after 24 h. At present, there are no clinical trials or guidelines that detail how we can manage these patients. Kim et al. examined the benefit of EVT in patients presenting very late. In their subgroup analysis of 150 patients who presented more than 16 h from their last known well time, EVT was performed only in 24 patients but a propensity matched analysis showed it was associated with increased odds of having favorable functional recovery at 3 months (adjusted OR, 11.08 (95% CI, 1.88–108.60). In a further subgroup of patients 24 h from last known well, EVT was associated with favorable outcomes as well (adjusted OR, 10.54; 95% CI, 2.18–59.34) (83). These preliminary studies substantiate the understanding that in patients with good collaterals, there is a chance to maintain the penumbra beyond 24 h.

### EVT in Posterior Circulation Strokes

There is much uncertainty related to treating posterior circulation stroke caused by basilar artery occlusion (BAO) with mechanical thrombectomy. The Basilar Artery International Cooperation study (BASICS) and endovascular treatment vs. standard medical treatment for vertebrobasilar artery occlusion (BEST) clinical trials are the only randomized clinical trials to date designed specifically to study the outcome of EVT in patients with basilar stroke within 6–8 h of onset (84, 85). However, the benefits of thrombectomy in the anterior circulation have not been replicated in basilar occlusions. These trials enrolled 300 patients and 131 patients respectively with more patients receiving IV tPA in the BASICS trial (80 vs. 30%). Favorable functional outcome was defined as mRS 0 to 3 at 90 days, and this occurred in 44.2% in the endovascular group and 37.7% in the medical care group (risk ratio, 1.18; 95% CI, 0.92 to 1.50) in the BASICS trial, and 42% in the endovascular group vs. 32% in the medical group (adjusted OR, 1.74; 95% CI, 0.81–3.74) in the BEST trial. In addition, SICH occurred in 4.5% of the patients after endovascular therapy and in 0.7% of those after medical therapy (risk ratio, 6.9; 95% CI, 0.9 to 53.0) in the BASICS trial and 8% in the endovascular arm and 0% in the control arm (*p* = 0.06) in the BEST trial. Nonetheless, it is worth noting that the investigators concluded that results of these trials may not exclude a substantial benefit of endovascular therapy as reflected by the wide confidence interval for the primary outcome. This is especially so considering that AIS secondary to BAO are often devastating for patients due to its high morbidity and mortality rates.

There are no randomized trials on thrombectomy in more distal posterior occlusions, and most of the pivotal trials excluded such patients. Strambo et al. examined the outcome of EVT in patient with isolated PCA occlusion vs. best medical therapy (BMT). They reported that complete recanalization at 24 h was achieved in 68% of patients undergoing EVT vs. 34.5% in BMT group (OR = 4.11; 95% CI = 1.35–12.53). This translated into a 15% absolute difference in the proportion of good outcome at 3 months in favor of the EVT group (55 vs. 40.5%), and a 25% absolute difference in visual field normalization at 3 months (50 vs. 25.4%) as well as a significantly better cognitive outcome with EVT (50 vs. 16.1%). In terms of complications, the frequency of SICH and 3-month mortality was similar in both groups (86). The Thrombectomy for Primary Distal Posterior Cerebral Artery Occlusion Stroke or TOPMOST study was a multicentric case-control propensity-matched studies for primary occlusion of the PCA treated with EVT. In 184 matched patients, the NIHSS decreased by a mean of 2.4 points at discharge in the medical group and 3.9 points in the thrombectomy group (mean difference, −1.5 points; 95% CI, 3.2 to −0.8; *p* = 0.06); this was balanced by an incidence of 4.3% SICH in both arms (87).

### Direct to Thrombectomy Table—No IV tPA

Time is an important variable that affects functional outcome in acute stroke thrombectomy and any delay in treatment initiation negatively impacts patients' functional outcomes (6).

While IV tPA has the ability to recanalize acute stroke occlusions, this has been eclipsed by the much superior recanalization rate of EVT. There is now a school of thought that instead of administering IV tPA, whether at an intervening primary stroke center or the comprehensive stroke center, patients with acute ischemic stroke from an LVO should go directly to endovascular thrombectomy. An RCT carried out in Japan entitled the SKIP trial comprised 200 AIS patients with anterior circulation occlusions presenting within 4 h of onset (88). At 3 months, the rate of good functional outcome was similar between the direct thrombectomy (59%) and combined bridging approach (57%). Furthermore, the mortality rate was similar between both arms as well. However, it was unable to prove noninferiority of direct to thrombectomy over bridging IV tPA (0.6 mg/kg Japanese standardized dose) because it was underpowered with a modest sample size. While the rate of asymptomatic hemorrhage was not significantly different between both arms and the rate of SICH was similarly non-significantly different, the combined rate of any ICH was significantly lower for the EVT group.

There were two other bridging IV tPA in thrombectomy trials which were similar in design to the SKIP trial but conducted across multiple stroke centers in China. The DIRECT-MT trial, in which 656 patients were enrolled, revealed that endovascular thrombectomy alone was non-inferior to combined intravenous alteplase and endovascular thrombectomy with regard to the functional outcome at 90 days (adjusted common odds ratio, 1.07; 95% confidence interval, 0.81 to 1.40; *p* = 0.04 for non-inferiority). Of note, the non-inferiority margin was set at a high value of 20% margin of confidence in this trial (89). In the DEVT trial, the non-inferiority test also demonstrated that the endovascular thrombectomy alone was non-inferior to the combined IV thrombolysis and endovascular thrombectomy group (*z* = 2.7157, *p* for non-inferiority = 0.003) (90). This trial was terminated after first interim analysis in May 2020 as outcome measured crossed the pre-specified efficacy boundary. In this study, there was no significantly different rate of symptomatic ICH between groups; however, the rate of any ICH is significantly higher in the bridging r-TPA and thrombectomy group.

It is also worth noting that these trials were performed in east Asian populations, and further evidence is needed in a more diverse population, the SWIFT-DIRECT, MR CLEAN NO-IV (91), and DIRECT-SAFE trials are upcoming international RCTs that can provide more information on the adoption of thrombectomy alone approach against bridging IV tPA (92).

### Stroke Secondary to Distal Medium Vessel Occlusion

Endovascular thrombectomy is an evidence-based, guideline-recommended treatment for acute ischemic stroke secondary to large vessel occlusion in the anterior circulation. However, endovascular treatment of DMVO is still unproven in view of the higher risk-benefit ratio with less severe clinical deficits and increased risk of iatrogenic complications. The exact definition of medium-sized vessel also requires consensus, as vessel size and anatomy may be subjected to interobserver variability, although a definition of medium vessel occlusion has been proposed using both anatomical characteristics and functional deficit (93).

Medium vessel occlusion was defined as occlusions of the M2/M3 middle cerebral artery/A2/A3 anterior cerebral artery and P2/P3 posterior cerebral artery segments in a study with pooled data from two multicenter prospective cohorts. In this study, only 50.0% of patients with DMVO achieved an excellent outcome (mRS score, 0–1) at 90 days and 67.4% achieved an independent outcome (mRS score, 0–2). The authors did find that intravenous alteplase was significantly associated with lower mRS scores in mRS shift analysis, but there was no significant association with excellent outcome (mRS score, 0–1). Moreover, even in the alteplase group, early recanalization was achieved in <50% of study cohort, suggesting insufficient efficacy of intravenous alteplase as a stand-alone treatment for DMVO strokes (94).

There is some preliminary evidence from a meta-analysis of data from 12 nonrandomized studies which suggested that endovascular thrombectomy for patients with occlusions of M2 segment of Middle Cerebral Artery that can be safely accessed is associated with high recanalization rates and good clinical outcomes (95). In addition, a more recent meta-analysis of data from the HERMES Collaboration showed that for patients with M2 occlusions, treatment effect favored EVT over control (adjusted OR, 2.39;, 95% CI, 1.08 to 5.28; *p* = 0.03) for mRS 0–2 at 90 days, with number needed to treat for one patient to have functional independence (mRS 0–2) being 5.4 (96). There are also several single-center studies which provided encouraging evidence on the effect of endovascular thrombectomy in treating DMVO (97, 98), and there is hope that reperfusion rates with EVT could improve further with the use of smaller diameter next-generation stent retrievers and aspiration devices. We also wish to emphasize that direct aspiration thrombectomy in distal vessels such as the M3 or M4 has a risk of avulsion injury to the perforators and further studies are needed to determine their safety and effectiveness.

### Tandem Occlusions

A tandem occlusion (TO), i.e., a thromboembolic obstruction in the intracranial cerebral vasculature in combination with an extracranial carotid artery occlusion, can occur in up to one-sixth of ischemic stroke patients (99). They tend not to have good recanalization rates with IV tPA and endovascular treatment is therefore advocated (100). In fact, subgroup analyses of the ESCAPE and MR CLEAN studies have suggested that patients with TO have better outcomes with early or concurrent treatment of the extracranial occlusion rather than later in a staged procedure (101, 102). Despite this, the optimal endovascular procedure in acute TO generally remains unclear. The controversy now is the optimal method of treating TO in acute stroke, i.e., is it better to initially bypass the extracranial occlusion and remove the intracranial occlusion first before returning to tackle the extracranial stenosis (the “retrograde” approach)? or is it preferable to attempt primary recanalization of the extracranial occlusion first, before moving on to treat the intracranial occlusion (the “antegrade” approach)?

The usual antegrade approach uses primary stenting to jail the extracranial stenotic atheromatous plaque, which should prevent showering of distal emboli (103). A theoretical drawback of the antegrade approach is the procedural time used for carotid stent placement which delays the time to intracranial reperfusion, which might result in an increase of the final infarct volume (104, 105). Furthermore, stent retriever-based thrombectomy techniques have the potential of entanglement between the struts of the stent retriever and carotid stent during withdrawal if the guiding catheter could not be advanced through the carotid stent. The retrograde approach achieves intracranial recanalization faster and some purport that this gives better functional outcomes (106); however, it may be difficult to pass through the proximal occlusion, and subsequent emboli from the proximal occlusion can sometimes re-occlude the intracranial circulation.

### Type of Anesthesia for Thrombectomy

Anesthesia support is necessary for patients undergoing EVT particularly in complex anatomy, difficult cases, or in restless aphasic patients. The modality of anesthesia may have significant implications in outcomes for mechanical thrombectomy and each has its own proposed benefits. General anesthesia (GA) may improve procedural safety by keeping the patient still and protecting the airway, while conscious sedation (CS) has the benefit of neurologic monitoring with hemodynamic stability, and a quicker puncture time (107). It has been difficult to determine which is better for thrombectomy as the literature is very heterogeneous. The initial retrospective studies often based their anaesthesia choice on patient characteristics and what the operator was comfortable with. A retrospective study of 1,174 patients from 2009 to 2013 concluded that GA was inferior to CS; however, there was limited data on the important factors such as blood pressure and the NIHSS; moreover, their outcomes studied were only mortality and length of stay (108). Conversely, another retrospective database study of 2,512 patients concluded the opposite: that CS was superior to GA for stroke interventions. However, it too had many important factors lacking (109).

In the more recent randomized trials attempting to address this topic, the GOLIATH (110), ANSTROKE (111), and SIETSA (112) trials reported GA and CS to be equally safe. In these trials, there was a prespecified target of systolic blood pressure of >140 mmHg prior to revascularization. A later analysis of these three studies showed that a blood pressure of 70 mmHg or less was associated with a significantly worse functional outcomes. GA in those same studies had a higher incidence of mean arterial pressure decreases of 20% or more (113). The worse outcomes of GA with EVT may be explained by decreases in blood pressure and there may be a need to maintain the blood pressure when choosing the type of anesthesia. A more updated meta-analysis which included studies up to 2020 seems to support the RCT findings. This meta-analysis looked at 1,711 subjects undergoing GA and 1,961 subjects using CS. They found no significant difference between the two modalities for functional outcomes, recanalization, mortality, or complications although there was a trend toward SICH for GA (114).

Finally, local anesthesia alone without sedation is emerging as a possible alternative to conscious sedation. In a meta-analysis of 7,797 patients, there was no difference in functional outcomes between GA and LA or between CS and LA; however, there was a trend toward excellent functional outcome (mRS ≤ 1) in the LA group vs. the GA group (OR = 1.44; 95% CI, 1.00 to 2.08; *p* = 0.05; *I* = 70%) and a trend toward improved mortality in the LA group vs. the GA group (adjusted OR = 1.24; 95% CI, 1.00 to 1.54; *p* = 0.05; *I* = 0%). The authors conclude that further anesthesia trials should have LA analyzed as a separate arm (115).

## Conclusion

The field of thrombectomy in acute stroke continues to evolve rapidly, and periodic reviews of the literature are important. We present several of the evidence-based improvements to the procedure and pertinent issues that require further data to settle the controversy.

## Author Contributions

MJ was involved in data gathering, data analysis, drafting of the article, and is agreeable to be accountable for all aspects of the work. BT, PB, AG, CY, TT, FA, C–HS, SH, and TA was involved in the drafting of the article and the final approval. LY was involved in the conception and design of the project, data gathering, data analysis, drafting of the article, the final approval, and is agreeable to be accountable for all aspects of the work. All authors contributed to the article and approved the submitted version.

## Funding

LY has received substantial grant funding from the National Medical Research Council (NMRC), Singapore and substantial support from the ministry of health (MOH), Singapore.

## Conflict of Interest

TA is a consultant for Ablynx, Amnis Therapeutics, Medtronic, Cerenovus/J&J, Rapid Medical and Anaconda. AG is a consultant for Stryker, Medtronic, Penumbra and has received educational grants from Abbott. PB is consultant for Phenox. The remaining authors declare that the research was conducted in the absence of any commercial or financial relationships that could be construed as a potential conflict of interest.

## Publisher's Note

All claims expressed in this article are solely those of the authors and do not necessarily represent those of their affiliated organizations, or those of the publisher, the editors and the reviewers. Any product that may be evaluated in this article, or claim that may be made by its manufacturer, is not guaranteed or endorsed by the publisher.
